# Visualization of Early Events in Acetic Acid Denaturation of HIV-1 Protease: A Molecular Dynamics Study

**DOI:** 10.1371/journal.pone.0019830

**Published:** 2011-06-29

**Authors:** Aditi Narendra Borkar, Manoj Kumar Rout, Ramakrishna V. Hosur

**Affiliations:** 1 Institute of Bioinformatics and Biotechnology, University of Pune, Ganeshkhind, Pune, India; 2 Department of Chemical Sciences, Tata Institute of Fundamental Research, Homi Bhabha Road, Colaba, Mumbai, India; 3 UM-DAE Centre for Excellence in Basic Sciences, Mumbai University Campus, Kalina, Santa Cruz Mumbai, India; Aston University, United Kingdom

## Abstract

Protein denaturation plays a crucial role in cellular processes. In this study, denaturation of HIV-1 Protease (PR) was investigated by all-atom MD simulations in explicit solvent. The PR dimer and monomer were simulated separately in 9 M acetic acid (9 M AcOH) solution and water to study the denaturation process of PR in acetic acid environment. Direct visualization of the denaturation dynamics that is readily available from such simulations has been presented. Our simulations in 9 M AcOH reveal that the PR denaturation begins by separation of dimer into intact monomers and it is only after this separation that the monomer units start denaturing. The denaturation of the monomers is flagged off by the loss of crucial interactions between the α-helix at C-terminal and surrounding β-strands. This causes the structure to transit from the equilibrium dynamics to random non-equilibrating dynamics. Residence time calculations indicate that denaturation occurs via direct interaction of the acetic acid molecules with certain regions of the protein in 9 M AcOH. All these observations have helped to decipher a picture of the early events in acetic acid denaturation of PR and have illustrated that the α-helix and the β-sheet at the C-terminus of a native and functional PR dimer should maintain both the stability and the function of the enzyme and thus present newer targets for blocking PR function.

## Introduction

Protein folding/unfolding, dynamics and denaturation play crucial role in cellular processes and thus have been the subject of extensive investigation for the last several decades. A variety of experimental and theoretical approaches have been employed to explore various aspects of the processes. While these have led to some generalized concepts, the mechanistic details remain largely unclear. They can vary from system to system - which is perhaps not too surprising – and residue level details are very seldom available.

The HIV-1 protease (PR) is a homodimeric aspartate protease that plays sensitive function in HIV-1 maturation. Hence, it is the subject of extensive pharmaceutical research with strategies encompassing blocking of both PR function and structure dynamics [1], [2]. Thus, both the native state and the denaturation dynamics of PR become crucial topics of study to answer the structure-to-function relation in PR. While there have been many experimental studies on the structure and dynamics of PR complexed to many inhibitors, reports on free protease are rather few because of the autolytic nature of the protease, and the number of reports on the denatured states is even less. Previous studies by NMR [3], [4], [5] and fluorescence experiments [6] have shown that the denatured states of PR created by different denaturants are far from random coils and contain elements of both native and non-native structures. Other experimental analyses of the ‘unfolded’ state of PR even under strong denaturing conditions have also revealed presence of transient folding nuclei (FN) that include residues spanning the active site, the hinge region, and the dimerization domain [7]. Using NMR investigations, acid denatured states [8] have been shown to be significantly different from the guanidine denatured tethered dimer of PR [9] or urea denatured PR precursor having the TFR extension at the N-terminal [10].

However, all these experimental studies can only allude to the regions of the PR that are structured or denatured but cannot provide direct structural visualization of the dynamics of the protein as a whole in the native or denaturing environments. Such visualization is however possible with theoretical techniques like all-atom molecular dynamics simulations (MD) and plethora of other secondary structure calculation algorithms. This provides the motivation for taking up extensive theoretical studies for understanding protein structure dynamics and denaturation.

Many MD studies on PR mechanism [11]–[13], drug resistance [14]–[16] and unfolding [17]–[21] have already been reported. While the mechanism and drug resistance simulations generally employ liganded PR as the starting structure, the unfolding simulations were performed with higher temperature as the denaturing condition. Extensive all-atom 100 ns simulation of both the monomeric and dimeric PR has shown greater flexibility of the termini in the monomer [17] and decoupling between monomer folding and dimerization [18]. Simulation of the 99-residue monomer in water at different temperatures revealed early assembly of the N and C termini into stable β-sheet structures [19]. These studies emphasize that PR is not a two-state dimer, as indicated by equilibrium denaturation experiments, but a three-state dimer with a marginally stable monomeric intermediate which involves the swapping of the flexible termini across the two chains to form the dimer interface. Apart from the termini, the ‘flaps’ too have been shown to be highly flexible regions of the PR [20], [21]. The flap dynamics is considered to be essential in target entry and exit from the PR active site and corresponding open and closed conformations are observed in the crystal structures. MD simulation has also revealed that the unliganded protease predominantly populates the semi open conformation, with closed and fully open structures being a minor component of the overall ensemble and also provides a model for such flap opening and closing [20].

All these all-atom MD simulations of PR dealt with gross structure dynamics only and did not present a consolidated picture of the denaturation process, sequential loss of residue level interactions etc. Thus, in this study, we have derived this information using all-atom molecular dynamics simulation of PR dimer and monomer in acetic acid and water environments separately. For illustration we have chosen here acetic acid denaturation, but such studies can be extended to denaturation by other agents as well.

## Results and Discussion

### 1. Denaturation of the dimeric PR

**Figure 1:**
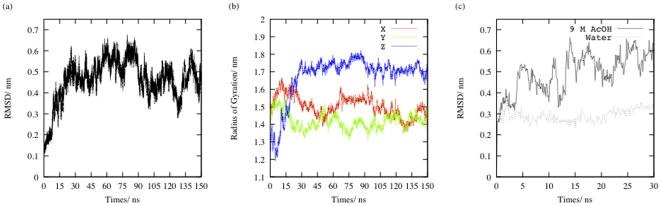
The root mean square deviation (RMSD) of the backbone atoms in the trajectory of the (a) dimer and (c) monomer in 9 M AcOH from the backbone atoms of the NMR structure of PR and (b) the radii of gyration of PR separately about the x, y and z axes during the 150 ns simulation of dimeric PR in 9 M AcOH. The RMSD in 9 M AcOH does not equilibrate and fluctuates more than 10 Å indicating a non-equilibrium run.

Due to the presence of high concentration of acetic acid molecules, the simulation is expected to be a non-equilibration run and the root mean square deviations (RMSD) of the backbone atoms of the dimeric PR trajectory with respect to the initial structure [Figure 1(a)] appropriately shows large continuous fluctuations of more than 0.1 nm and do not converge. The RMSD value shows a steep immediate ascent to 0.35 nm from 0.2 nm at around 10 ns. Following this, it rises further up to 0.6 nm and fluctuates substantially but never falls below the 0.3 nm mark. Contrary to this, in the simulation of the dimer in water, soon after 1 ns, the RMSD fluctuates only between 0.25–0.3 nm and thus the structure can be said to have quickly converged in the water simulation [Figure S1]. Thus, at around 10 ns during the acetic acid simulation, some sudden structural change in the PR conformation shifts it away from the native state and then the PR fluctuates between different non-native states.

To locate this structural transition, we first looked at the values of radius of gyration (R_G_) of the dimeric PR about its x, y and z Cartesian coordinate axes separately. These three different values give a global indication of the shape of the molecule and their fluctuations represent the extent of flexibility along the corresponding axis of the molecule. Figure 1(b) very clearly reveals that during the first 25 ns of simulation in 9 M AcOH the dimeric PR steadily elongates along the z axis [Figure S2] and so the two monomer units in the PR dimer should move away from each other during this initial phase of dimer denaturation in 9 M AcOH.

**Figure 2:**
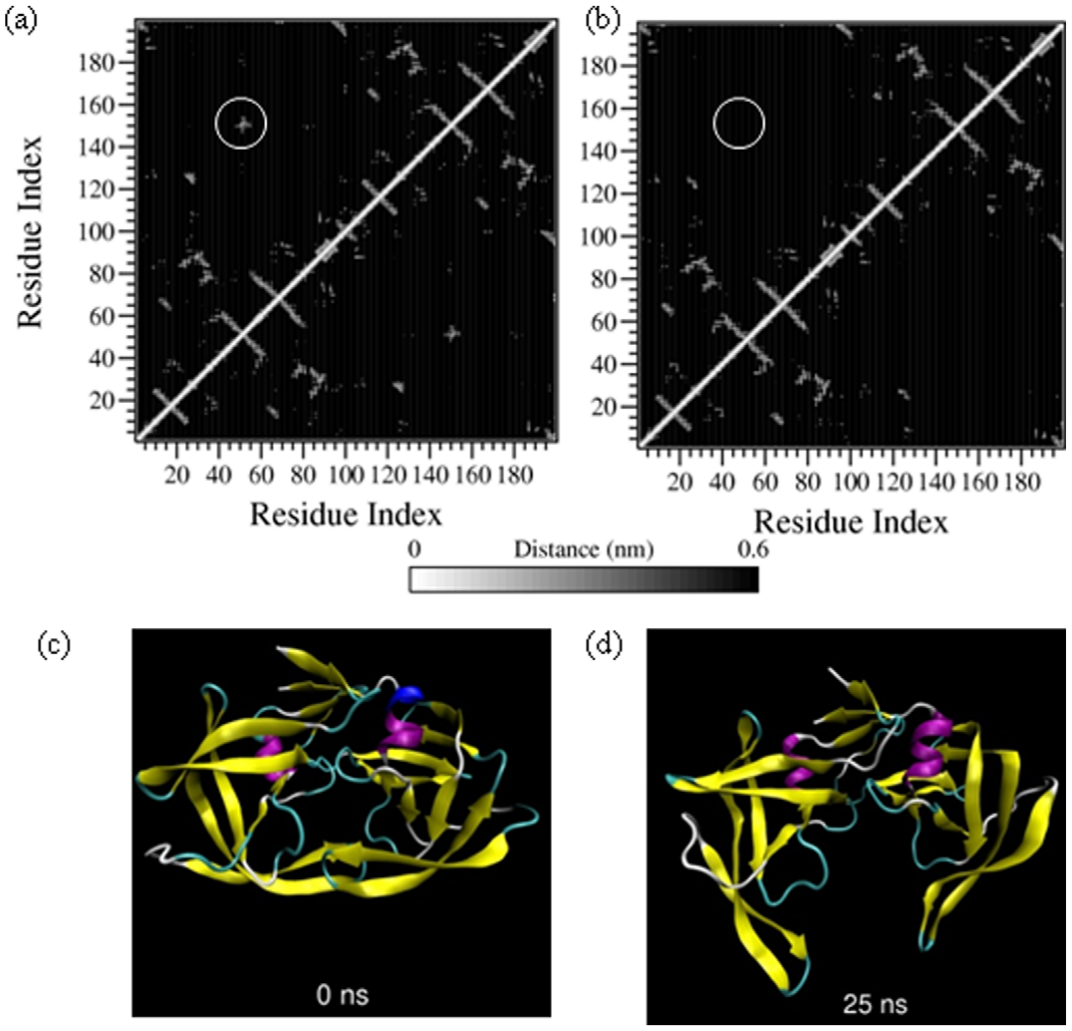
Tertiary contacts at (a) 0 ns and (b) 25 ns and snapshot structures at the same time points i.e. at (c) 0 ns and (d) 25 ns of the simulation of dimeric PR in 9 M AcOH. The circled region denote the tertiary contacts between the protein flaps in the 0 ns structure and complete absence of these contacts in the 25 ns structure. Corresponding snapshot figures illustrate the marked separation of the protein flaps and the catalytic triads of the monomeric units. For detailed description of the tertiary contact profiles, refer to supplementary material S4.

To confirm this movement, we next looked at the tertiary contact profile of the trajectory. A tertiary contact is defined between two residues when the non-bonded heavy atoms of the residues lie within 0.6 nm of each other [Figure S3]. Figures 2(a) and 2(b) show the tertiary contact profile of the initial structure and 25 ns snapshot from the 9 M AcOH trajectory of the dimeric PR. The latter figure has clear absence of any tertiary contact between the flaps (residues 45 to 55 in both monomeric units) of the dimeric PR. It also reveals reduced contacts between the catalytic triad (residues 25 to 27) of both the monomeric units; whereas the β-sheet formed by the N and C terminal of the two monomers seems to remain intact. Thus, the dimer seems to elongate in such a fashion that the monomer units separate at the flaps but remain tethered by the N and C terminal β-sheet [Figures 2(c) and 2(d)]. Such movement can be clearly visualized in the movie file [Video S1] provided as supporting video with this article.

The hydrogen bond network (Fireman's Grip) between the catalytic triad of both the monomers in the PR is crucial for its function and so its disruption could lead to loss of PR function. We looked at this hydrogen bond network during the simulation of dimeric PR in 9 M AcOH [Figure S4] and this data indicates that the hydrogen bond network completely breaks by 25 ns.

Thus, by summing up these observations from the simulation of dimeric PR in 9 M AcOH, it can be concluded that as early as within the first 25 ns of the simulation, the monomeric units of the PR start separating at the flaps but remain tethered at the terminal β-sheet. This separation leads to disruption of the Fireman's grip and the PR becomes dysfunctional. Even up till further 150 ns, neither the monomers move closer nor the Fireman's grip gets reconstructed. Hence, the dimeric PR can be considered to be denatured and it is only a matter of a little more time when the terminal β-sheet will break and the dimer will truly separate into two monomers. Since the monomer folding and dimerization is reported to be decoupled [18], it can also be assumed that during the time required for the dimer to separate into monomers, the individual monomers will not denature. Consequently, we proceeded with simulation of the native PR monomer under similar conditions to probe further into the denaturation process.

### 2. Denaturation of the monomeric PR

**Figure 3:**
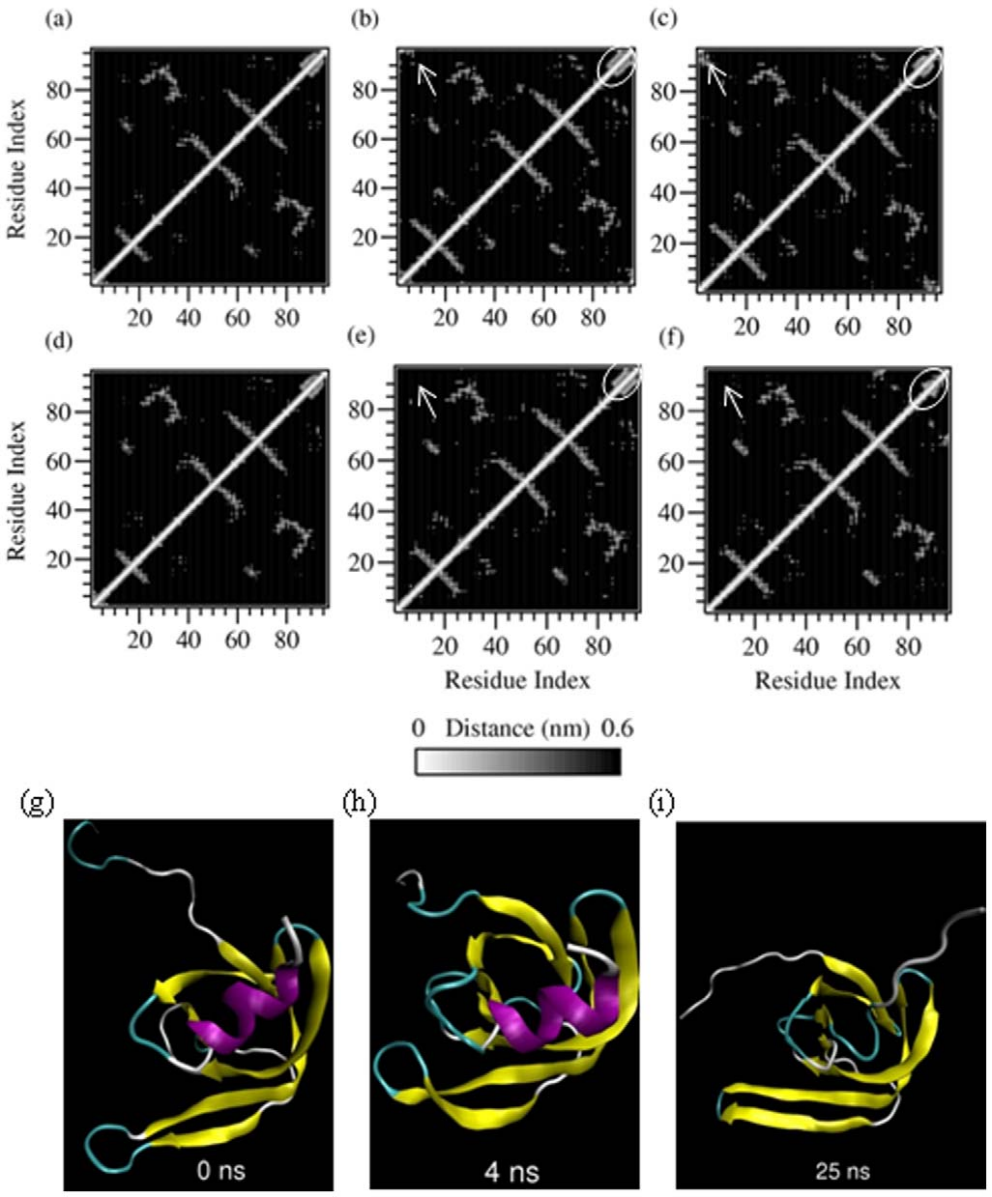
Tertiary contacts between the residues of dimeric PR simulated at (a) 0 ns, (b) 4 ns and (c) 25 ns in water and the corresponding tertiary contacts (d), (e) and (f) and trajectory snapshots (g), (h) and (i) in 9 M AcOH. Arrows indicate the interactions of the N and C termini that are seen in the simulation in water but not in the simulation in 9 M AcOH. The circled region indicates the α-helical interactions that completely vanish by 25 ns in the monomer simulation in 9 M AcOH.

The RMSD for simulation of monomeric PR in 9 M AcOH and water is given in Figure 1(c). For the initial 4 ns, both simulations show similar RMSD trend; but after this time point, the RMSD of the trajectory in acetic acid shows large fluctuations whereas the RMSD of the trajectory in water converges. Thus, again some conformational transition around 4 ns seems to prevent the structure from equilibrating in the acetic acid simulation. This transition was deciphered from the tertiary contact profiles at this time point [Figure 3]. Although the initial tertiary contact profiles at 0 ns for both the simulations in water [Figure 3(a)] and 9 M AcOH [Figure 3(d)] are similar; as we reach 4 ns, the N and C termini of the PR monomer in water start forming contacts [Figure 3(b)] whereas in the profile for acetic acid simulation, these contacts are distinctly absent [Figure 3(e)]. Thus, the β-sheet structure formed by the N and C termini of the PR must confer some structural stability to the protein and in the absence of these interactions the monomer trajectory gets propelled into a very random non-equilibrium path on the conformation landscape.

**Figure 4:**
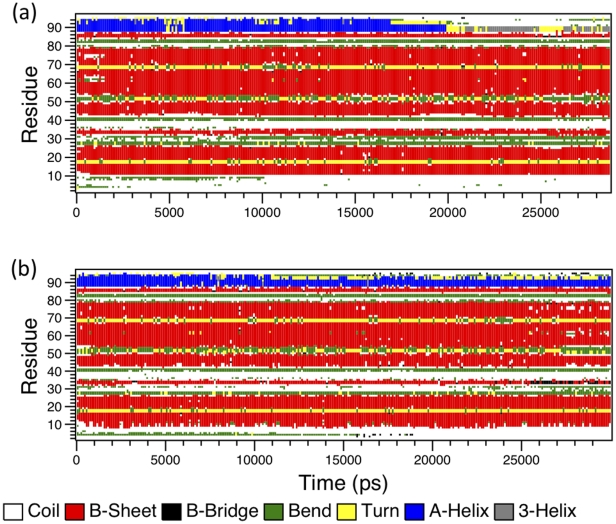
The secondary structure profile calculated using DSSP [**22**] for the initial 30 ns of the two simulations of monomeric PR a) in 9 M AcOH and b) in water.

Further, secondary structure calculation on the two trajectories [Figure 4(a) and 4(b)] reveals that even in the denaturing 9 M AcOH conditions, much residual secondary structure mainly as the β-sheet remains in PR. However, the C-terminal α-helix transits to a turn within 20 ns simulation of the PR in 9 M AcOH [Figure 3(d) to 3(i) and Figure 4(a)] but not in the water simulation [Figure 3(a) to 3(c) and Figure 4(b)] and neither during the full 150 ns of the dimer simulation in acetic acid [refer to the movie Video S1 and S2 provided as supporting videos with this article]. Gō model [2] simulations have indicated that the (post critical) folding nuclei of PR are or folding nucleus of PR is formed as a spatially closed unit of α helix (83–92) with sheet (74–78) above and another β-strand with sheet (24–34) perpendicular to these elements which essentially coincides with the highly protected structural units and the stabilization core defined by Wallqvist et al. [23]. Thus, the disruption of the α-helical structure and the consequent and persistent loss of the FN interactions cause the PR to denature. It is also interesting to note that lattice model calculations [24], [25] have shown a hierarchical model of PR monomer folding with docking of fragments (83–92) and (24–34) being the first step followed by relaxation of the remaining amino acids in the native structure shortly afterwards. In the unfolding process seen in our simulation, these fragments are also the first to dissociate and start the denaturation of the PR.

### 3. Insights into the mechanism of denaturation of PR by acetic acid

**Figure 5:**
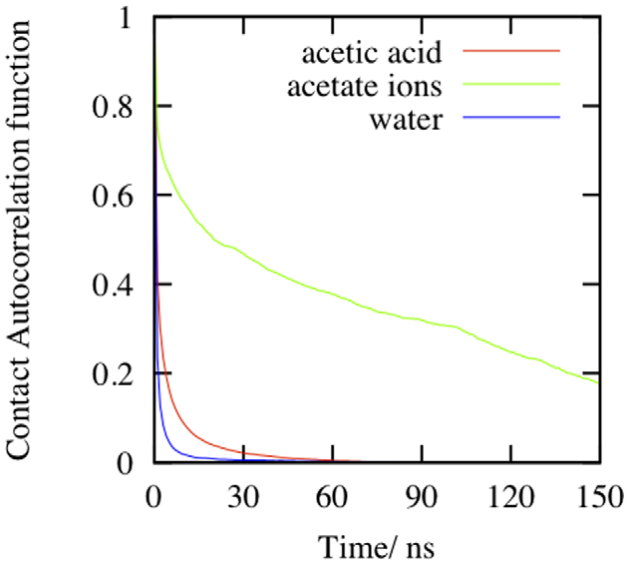
Contact autocorrelation function of acetate ions, acetic acid and water during the simulation of the PR monomer.

We next speculated about the driving force for such major structural transitions in the simulation of PR in acetic acid in such a short time period. Figure 5 shows the contact autocorrelation function of acetic acid and water with the PR during the simulation and Table 1 details the integrated correlation time calculated from these graphs. It is clear that acetate ions spend a lot of time on average around the PR, followed by neutral acetic acid molecules. Thus, the acetic acid species interacts with the PR more than water in the simulation.

**Table 1:** Mean residence time of acetate ions, acetic acid and water around the PR calculated by integrating the contact autocorrelation function for these chemical species during the simulation of the PR monomer in 9 M AcOH.

S. No.	Chemical Species	Mean Residence Time
1.	Acetate Ions	2158 ps
2.	Neutral Acetate Acid Molecules	373 ps
3.	Water Molecules	156 ps

**Figure 6:**
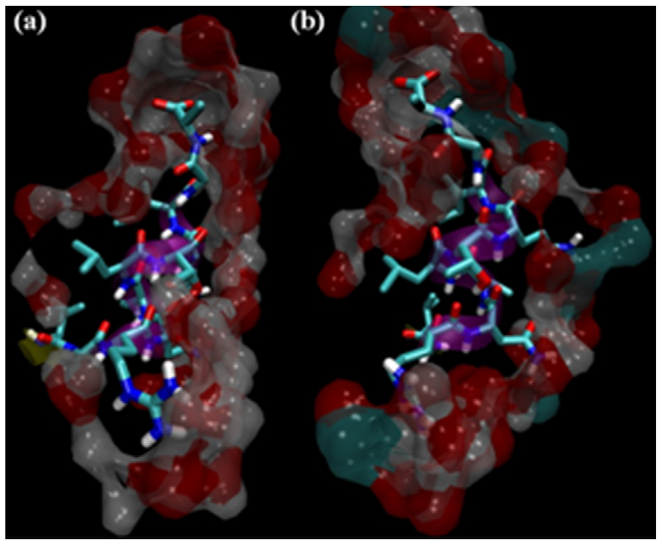
Envelope of solvent (a) water and (b) 9 M AcOH surrounding residues 85 to 95 of PR. This stretch contains the α-helix seen as purple ribbon in the figure. The envelope is drawn as a surface comprising of all solvent atoms that are within 3.5 Å of the residues 85 to 95.

Further, Figure 6 clearly illustrates the regions in PR where these molecules specifically reside. It clearly shows a continuous envelope of the solvent around the protein stretch from residues 85 to 95 which contain the alpha helix. This stretch also contains only three hydrophilic amino acids (R87, N88 and Q92) and one polar residue (T91) while all the remaining seven residues are hydrophobic. The acetic acid environment (with dielectric constant of 6.82) is amenable to these hydrophobic residues as well and they easily make contacts with the solvent envelope around them. Such new interactions overcome the scanty α-helical interactions easily, and residues 85 to 95 adopt random coil structure. In the highly polar environment of water (dielectric constant 80), this stretch of residues tries to minimize its interaction with the solvent envelope and thus maintains a compact alpha helix.

A similar argument could be extended to explain the stability of the β sheet core domain. The solvent molecules do not penetrate the core domain [Figure S5] to disrupt the hydrophobic interactions in this region and the solvent envelope outside the core domain does not furnish enough interactions to overcome both the extensive hydrogen bonding network and hydrophobic interactions in the β sheets of the core domain.

Thus, we have presented here the first direct visualization of the initial events in the denaturation of mature and functional PR in acetic acid using all atom Molecular Dynamics simulation in explicit solvent. Novel observations from these simulations have helped us to obtain a picture of the initiation of acetic acid denaturation of PR. The dimeric PR begins to denature by separating into monomers. Though the dimer interface and the critical hydrogen bond network of the catalytic triad of PR disrupt within first 25 ns of the simulation, the monomers remain tethered together by the β-sheet formed by the N and C termini for a longer duration of time. However, after separation into monomers, the folding nucleus formed by the α-helix and the surrounding β-strands is the first to disrupt during the denaturation of the monomer. Following the loss of these critical interactions, the PR follows a random and non-equilibrating path on the conformation landscape. Apart from this, we have also been able to look into the mechanism of denaturation by acetic acid and have attributed it to the direct interactions of acetic acid molecules with some sensitive regions of the protein such as the α-helical residues.

However, during the time duration monitored in this study, only some early events could be observed. Nevertheless, these provide satisfactory evidence of initiation of denaturation. Simulations up to microseconds may be required to visualize collapse of the robust β-sheet structure and complete denaturation of the protein. Such studies can also be extended to denaturation by other agents to obtain insights into the overall denaturation landscape of the protein. We believe that the observations reported in this study pinpoint the early and crucial events that can disrupt both the function and structure of the PR and hence can provide a basis for the design of newer targets for blocking PR function.

## Methods

### 1. Construction of 9 M acetic acid model

Both uncharged and charged acetic acid molecules were constructed using ArgusLab software [http://www.arguslab.com] and their parameters were obtained from PRODRG server at Dundee [26] [http://davapc1.bioch.dundee.ac.uk/prodrg/]. The uncharged molecule was duplicated and a box [5 Å×5 Å×5 Å] containing 334 molecules of acetic acid was constructed using Vega ZZ 2.3.2 [27] [http://www.ddl.unimi.it]. All the further steps in setting up of the model were then carried by GROMACS 4.0.5 [28], [29] tool and all MD simulations were performed in periodic boundary conditions using GROMACS 4.0.5 tool with GROMOS96 force field [30]. The acetic acid box was solvated with SPC216 water molecules [31]. The ratio of number of acetic acid molecules to that of water in the solvated box was adjusted to 1∶3 and the density was adjusted to 1.02 kg l^−1^ such that this box models 9 M AcOH. Since the force field parameters for acetic acid molecules obtained in the GROMACS topology from PRODRG server and those for the SPC216 water molecules are already validated, we used these same validated force field parameters for the simulation of the 9 M AcOH solvent. After an initial energy minimization by steepest descent algorithm with tolerance of 100 kJ mol^−1^ nm^−1^ to remove any bad contacts, an MD run was set up for 1 ns to completely mix and equilibrate the contents of the box. Complete mixing and equilibration of the contents was confirmed by convergence of potential energy of the system and by convergence of the number of hydrogen bonds between acetic acid and water molecules using GROMACS 4.0.5 analysis tools.

### 2. Model preparation and MD setup for studying denaturation of PR

The crystal structure of mature PR dimer reported by Pillai et al. [32] (PDB ID 1G6L) and the solution structure of PR monomer reported by Ishima et al. [33] (PDB ID 1Q9P) was adopted as the starting structure. These were separately placed in a cubic box and solvated with the equilibrated 9 M AcOH solution and SPC216 water. The peripheral aspartates, glutamates, histidines and C-terminal of the PR were protonated to model the effect of protonation of the amino acids in low pH conditions (∼1.9) in 9 M AcOH and thirteen random acetic acid molecules were replaced with acetate ions (charged acetic acid molecules) to neutralize the additional charge gained by the system.

All models were energy minimized using steepest descent until they converged to 10 kJ mol^−1^ nm^−1^. A 50 ps MD dynamics run was then performed at 300 K with position restraint on the PR atoms. Finally a 150 ns MD was performed on the system of dimeric PR in 9 M AcOH box and a 30 ns MD was performed on the system of monomeric PR in 9 M AcOH and on monomeric and dimeric PR in water. All trajectory analyses were carried out by the analysis tools in GROMACS 4.0.5 package and VMD [34] [http://www.ks.uiuc.edu/Research/vmd/] was used for visualization purposes.

## Supporting Information

Figure S1 The root mean square deviation (RMSD) of the backbone atoms in the trajectory of the dimer in water from the backbone atoms of the NMR structure of PR.
(TIFF)

Figure S2 The definition of the x, y and z axis of the dimeric PR.
(TIFF)

Figure S3 The tertiary contact profiles for a PR dimer. The whole profile can be broken into four quadrants; the lower left and upper right quadrants represent the tertiary contacts within each monomer. The remaining two quadrants contain the interactions between the monomers. The boxed-off values are interactions between the different β-strands; circled values are the α-helical interactions; the values enclosed by a triangle represent interactions between the loops and β-strands; those enclosed by a box with rounded edge represent interactions between the loops and residues of the α-helix and arrows indicate the interactions at the dimer interface.
(TIF)

Figure S4 The hydrogen bond profile for the catalytic triad during the simulation of dimeric PR in 9 M AcOH. In the panels for monomer units, the first and second H-bonds form a part of the Fireman's grip whereas in the panel for dimer, the 2^nd^, 5^th^, 11^th^ and 12^th^ H-bonds form a part of the Fireman's grip. This profile has been generated by keeping H-bond donor-hydrogen distance cutoff of 0.3 nm and angle cutoff of 120°^.^. Thus, even with these lenient cutoff values, it is clear that only 2 of the eight H-bonds persist continuously throughout the trajectory. The 4 H-bonds in the monomer units exist intermittently whereas the 5^th^ and the 12^th^ H-bond in the dimer panel completely vanishes by 20 ns. Thus, by this time, the Fireman's grip can be considered to be broken and it doesn't get restored in the remainder of the simulation.
(TIFF)

Figure S5 Envelope of solvent 9 M AcOH surrounding the core domain of PR. The envelope is drawn as a surface comprising of all solvent atoms that are within 3.5 Å of the residues.
(TIF)

Video S1 (9MAcOH-150 ns-PRdimer.wmv): The movie file for the 150 ns trajectory of dimeric PR in 9 M AcOH. The movie contains snapshot of every 300 ps of the simulation.
(WMV)

Video S2 (9MAcOH-30 ns-PRmonomer.wmv): The movie file for the 30 ns trajectory of monomeric PR in 9 M AcOH. The movie contains snapshot of every 100 ps of the simulation.
(WMV)
