# Supplementary figures and images for: Visualization of Early Events in Acetic Acid Denaturation of HIV-1 Protease: A Molecular Dynamics Study
Source: PLoS One. 2011 Jun 29;6(6):e19830. doi: 10.1371/journal.pone.0019830 (PMC3126794; doi:10.1371/journal.pone.0019830)

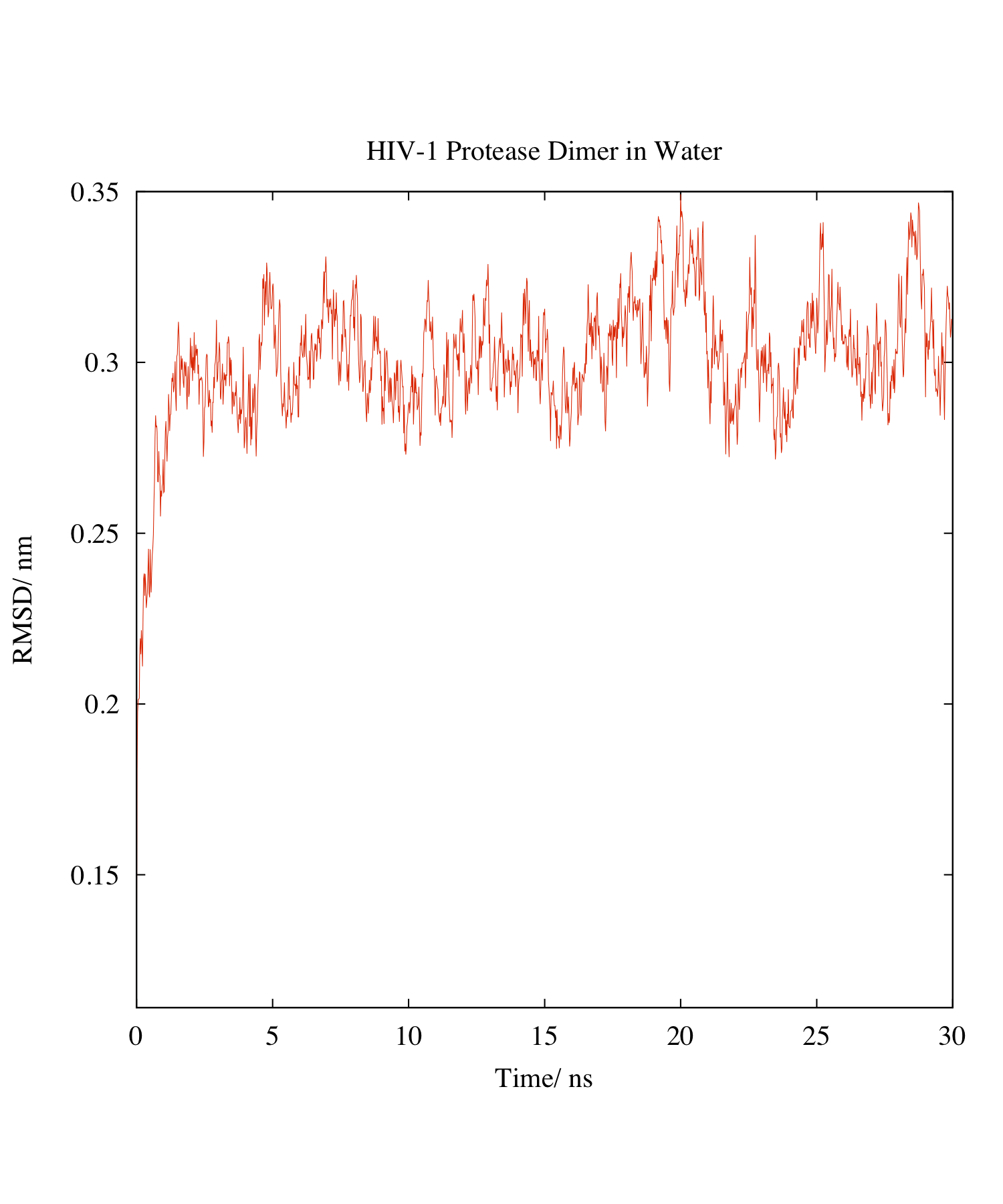

Supplement: Figure S1 — The root mean square deviation (RMSD) of the backbone atoms in the trajectory of the dimer in water from the backbone atoms of the NMR structure of PR. (TIFF) [file pone.0019830.s001.tif]

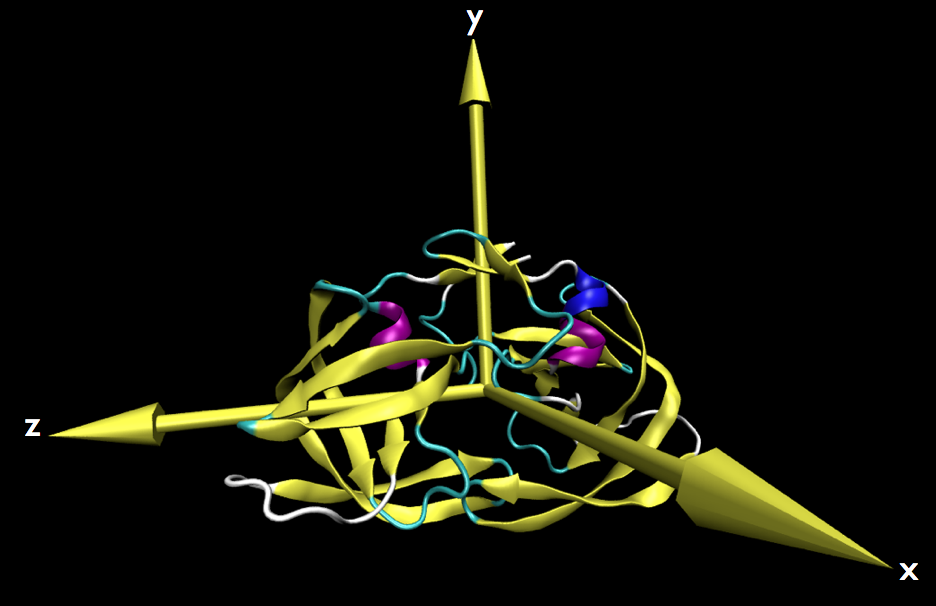

Supplement: Figure S2 — The definition of the x, y and z axis of the dimeric PR. (TIFF) [file pone.0019830.s002.tif]

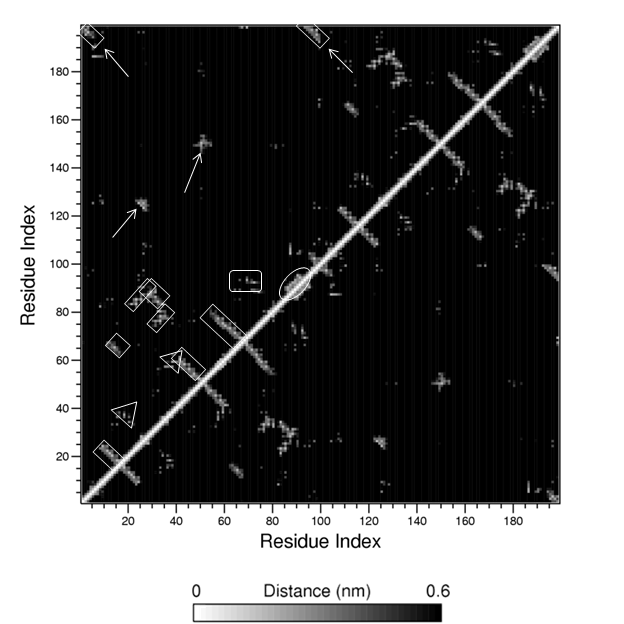

Supplement: Figure S3 — The tertiary contact profiles for a PR dimer. The whole profile can be broken into four quadrants; the lower left and upper right quadrants represent the tertiary contacts within each monomer. The remaining two quadrants contain the interactions between the monomers. The boxed-off values are interactions between the different β-strands; circled values are the α-helical interactions; the values enclosed by a triangle represent interactions between the loops and β-strands; those enclosed by a box with rounded edge represent interactions between the loops and residues of the α-helix and arrows indicate the interactions at the dimer interface. (TIF) [file pone.0019830.s003.tif]

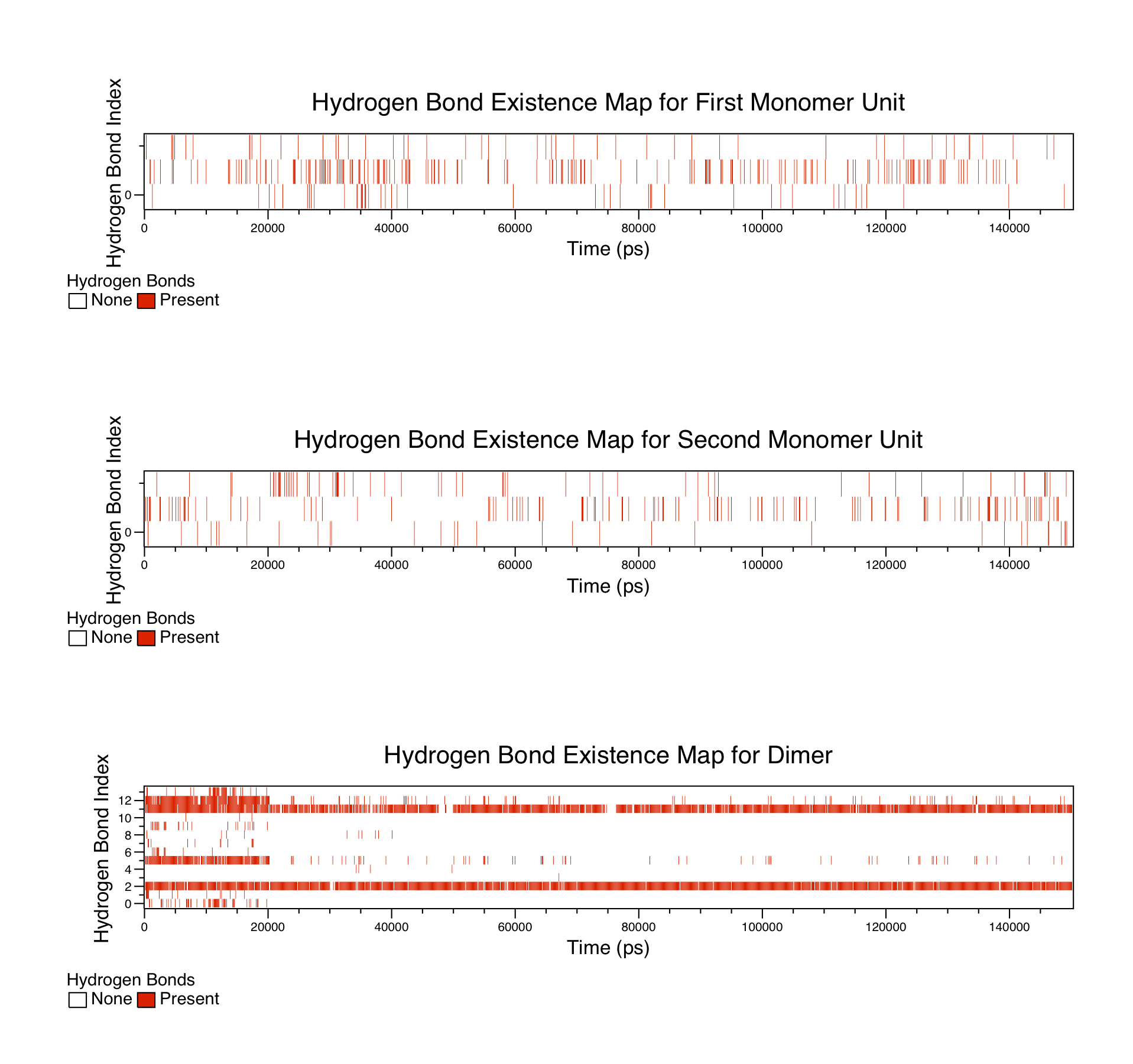

Supplement: Figure S4 — The hydrogen bond profile for the catalytic triad during the simulation of dimeric PR in 9 M AcOH. In the panels for monomer units, the first and second H-bonds form a part of the Fireman's grip whereas in the panel for dimer, the 2nd, 5th, 11th and 12th H-bonds form a part of the Fireman's grip. This profile has been generated by keeping H-bond donor-hydrogen distance cutoff of 0.3 nm and angle cutoff of 120°.. Thus, even with these lenient cutoff values, it is clear that only 2 of the eight H-bonds persist continuously throughout the trajectory. The 4 H-bonds in the monomer units exist intermittently whereas the 5th and the 12th H-bond in the dimer panel completely vanishes by 20 ns. Thus, by this time, the Fireman's grip can be considered to be broken and it doesn't get restored in the remainder of the simulation. (TIFF) [file pone.0019830.s004.tif]

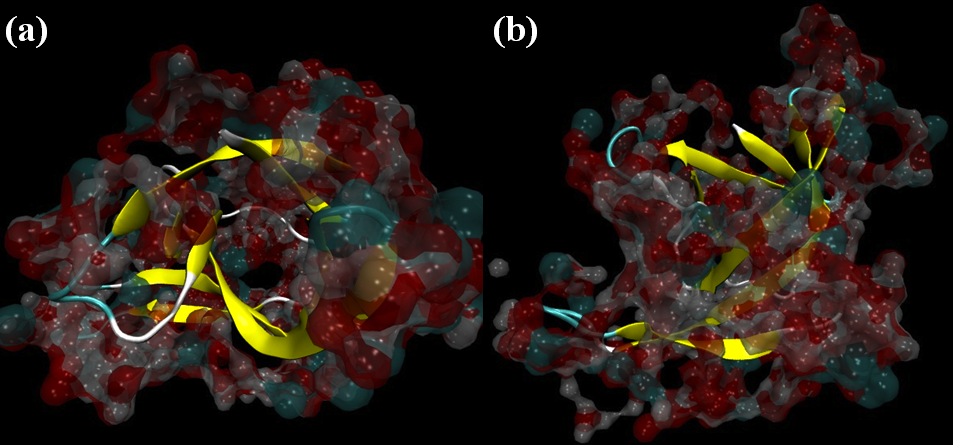

Supplement: Figure S5 — Envelope of solvent 9 M AcOH surrounding the core domain of PR. The envelope is drawn as a surface comprising of all solvent atoms that are within 3.5 Å of the residues. (TIF) [file pone.0019830.s005.tif]
